# Supplementary material for: Mechanism and regulation of cargo entry into the Commander endosomal recycling pathway
Source: Nat Commun. 2024 Aug 21;15:7180. doi: 10.1038/s41467-024-50971-0 (PMC11339278; doi:10.1038/s41467-024-50971-0)
Supplement: Supplementary file 1 — Supplementary Information [file 41467_2024_50971_MOESM1_ESM.pdf]

SUPPLEMENTARY INFORMATION

Mechanism and regulation of cargo entry into the Commander endosomal recycling pathway

Rebeka Butkovič[1,*], Alexander P. Walker[1,†], Michael D. Healy[2,†], Kerrie E. McNally[1,‡], Meihan Liu[2], Tineke Veenendaal[3], Kohji Kato[1], Nalan Liv[3], Judith Klumperman[3], Brett M. Collins[2,*], Peter J. Cullen[1,*].

[1]School of Biochemistry, Biomedical Sciences Building, University of Bristol, Bristol BS8 1TD, UK.

[2]Centre for Cell Biology of Chronic Disease, Institute for Molecular Biosciences, The University of Queensland, St. Lucia, QLD 4072, Australia.

[3]Center for Molecular Medicine, University Medical Center Utrecht, Institute of Biomembranes, Utrecht University, 3584 CX Utrecht, The Netherlands.

[†]equal contributions

[‡]present address: MRC Laboratory of Molecular Biology, CB2 0QH, Cambridge, UK

[*]co-corresponding authors: rebeka.butkovic@bristol.ac.uk; b.collins@imb.uq.edu.au; pete.cullen@bristol.ac.uk

This file contains 5 supplementary figures and figure legends and one supplementary table.

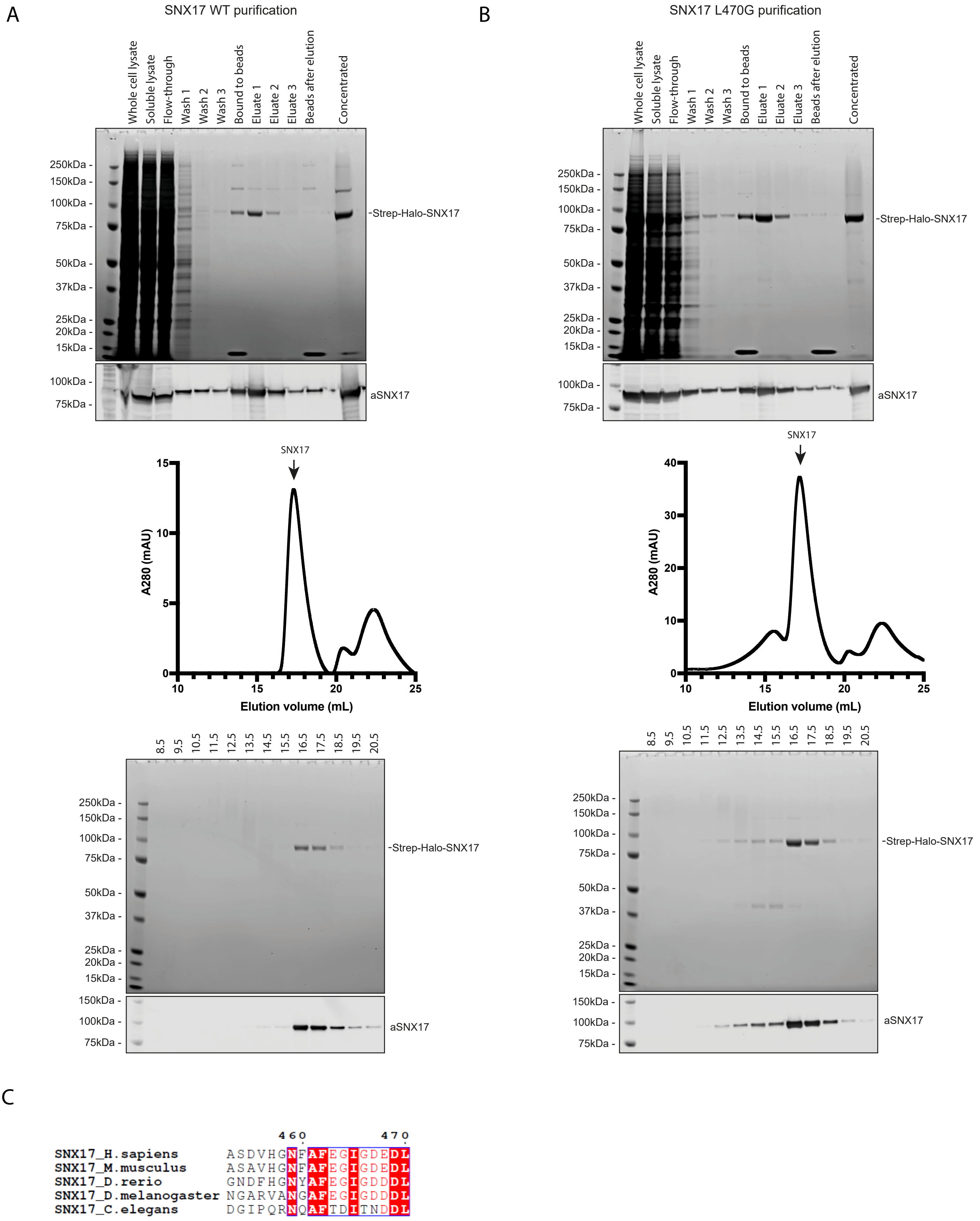
A
SNX17 WT purification
Whole cell lysate
Soluble lysate
Flow-through
Wash 1
Wash 2
Wash 3
Bound to beads
Eluate 1
Eluate 2
Eluate 3
Beads after elution
Concentrated
250kDa -
150kDa -
100kDa -
75kDa -
50kDa -
37kDa -
25kDa -
20kDa -
15kDa -
-Strep-Halo-SNX17
aSNX17
SNX17
A280 (mAU)
Elution volume (mL)
8.5 9.5 10.5 11.5 12.5 13.5 14.5 15.5 16.5 17.5 18.5 19.5 20.5
B
SNX17 L470G purification
C
460 470
SNX17_H.sapiens ASDVHGNFAFEGIGDEDL
SNX17_M.musculus ASAVHGNFAFEGIGDEDL
SNX17_D.rerio GNDFHGNYAFEGIGDDDL
SNX17_D.melanogaster NGARVANGAFEGIGDDDL
SNX17_C.elegans DGIPQRNQAFTDITNDDL

Supplementary Figure 1.

Purification of wild-type SNX17 (A) and SNX17(L470G) (B). (C) Evolutionary conservation of C-terminus of SNX17 was evaluated by aligning full length SNX17 protein sequences from different model organisms in Clustal Omega online tool and depicted using ESPript3.0. Last 18 residues from each species are shown. The residue numbering corresponds to human SNX17 protein.

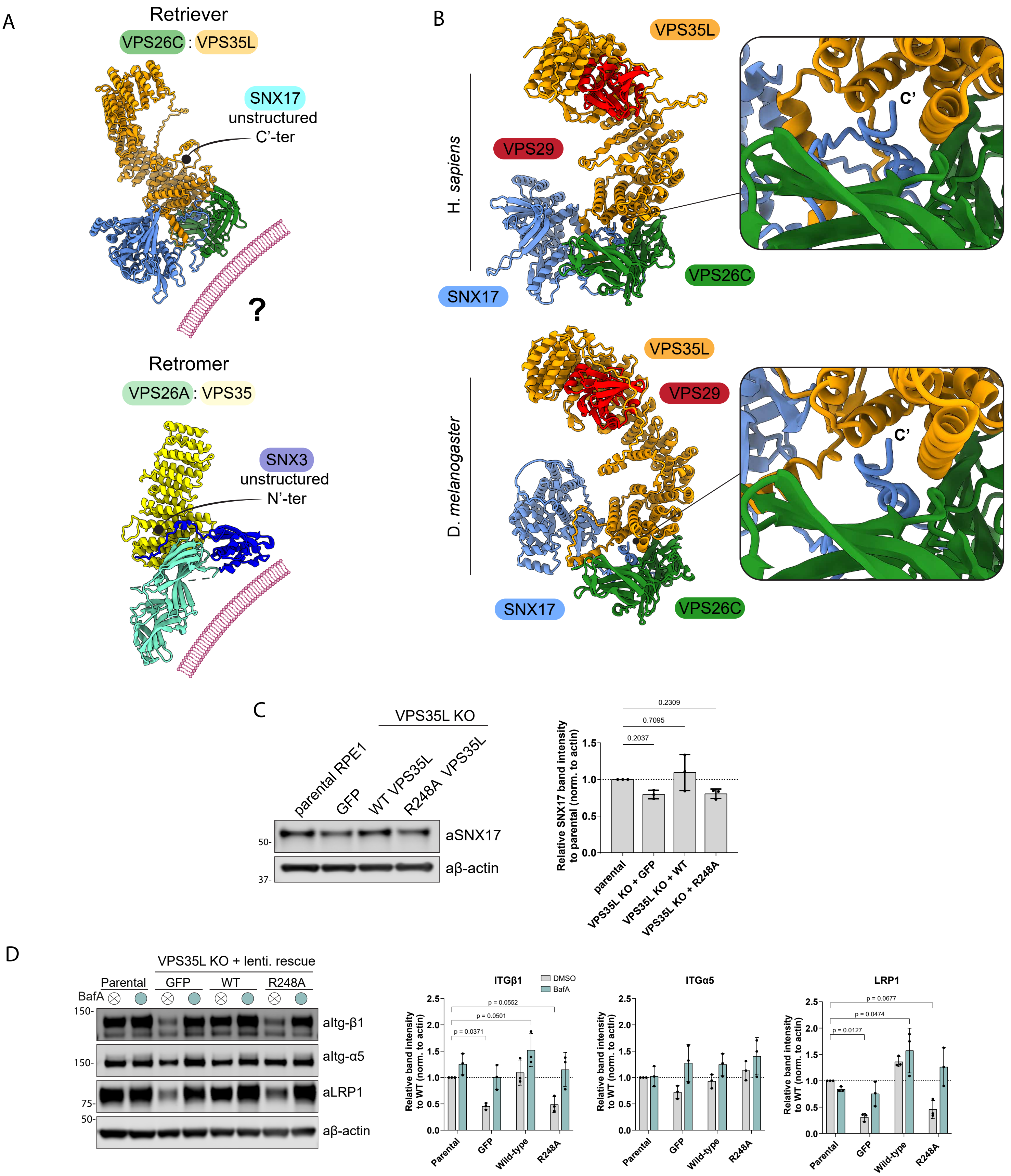
A
Retriever
VPS26C : VPS35L
SNX17
unstructured
C'-ter
?
Retromer
VPS26A : VPS35
SNX3
unstructured
N'-ter
B
H. sapiens
VPS35L
VPS29
SNX17
VPS26C
C'
D. melanogaster
VPS35L
VPS29
SNX17
VPS26C
C'
C
VPS35L KO
parental RPE1
GFP
WT VPS35L
R248A VPS35L
aSNX17
aβ-actin
50-
37-
Relative SNX17 band intensity to parental (norm. to actin)
0.2309
0.7095
0.2037
parental
VPS35L KO + GFP
VPS35L KO + WT
VPS35L KO + R248A
D
VPS35L KO + lenti. rescue
Parental
GFP
WT
R248A
BafA
150-
150-
75-
50-
aItg-β1
aItg-α5
aLRP1
aβ-actin
ITGβ1
DMSO
BafA
p = 0.0552
p = 0.0501
p = 0.0371
Relative band intensity to WT (norm. to actin)
Parental
GFP
Wild-type
R248A
ITGα5
LRP1
p = 0.0677
p = 0.0474
p = 0.0127

Supplementary Figure 2.

(A) Comparison of SNX17 binding to the VPS35L-VPS26C interface (AlphaFold2) and SNX3 binding to VPS35-VPS26A interface of the Retromer complex (X-ray, PDB 5F0J). Previously characterised membrane binding interface within retromer is depicted with the cartoon of phospholipid bilayer. The possible orientation of SNX17-Retriever complex relative to membrane is shown. (B) Comparison of AlphaFold2-predicted conformations of SNX17-Retriever complex in human and Drosophila emphasizes evolutionary conservation of the assembly. (C) VPS35L KO RPE1 cells were lentivirally transduced with GFP, VPS35L-GFP or VPS35L-GFP(R248A). Protein lysates were then resolved using immunoblot and whole-cell levels of SNX17 were compared in all samples. The quantification from 3 independent experiments is shown on the right. $n = 3$, 1-way ANOVA with Dunnett's multiple comparison test, error bars represent s.d. (D) Parental RPE1 cells and VPS35L KO RPE1 cells lentivirally transduced with GFP, VPS35L-GFP or VPS35L-GFP(R248A) were treated with either DMSO or 100 nM Bafilomycin A for 18h to prevent lysosomal degradative activity. Protein lysates were then resolved for western blot analysis and whole-cell levels of cargo proteins (Itg-α5, Itg-β1 and LRP1) and β-actin were compared in all samples. 2-way ANOVA with Dunnett's multiple comparison test, error bars represent s.d., only changes with $p < 0.1$ are shown.

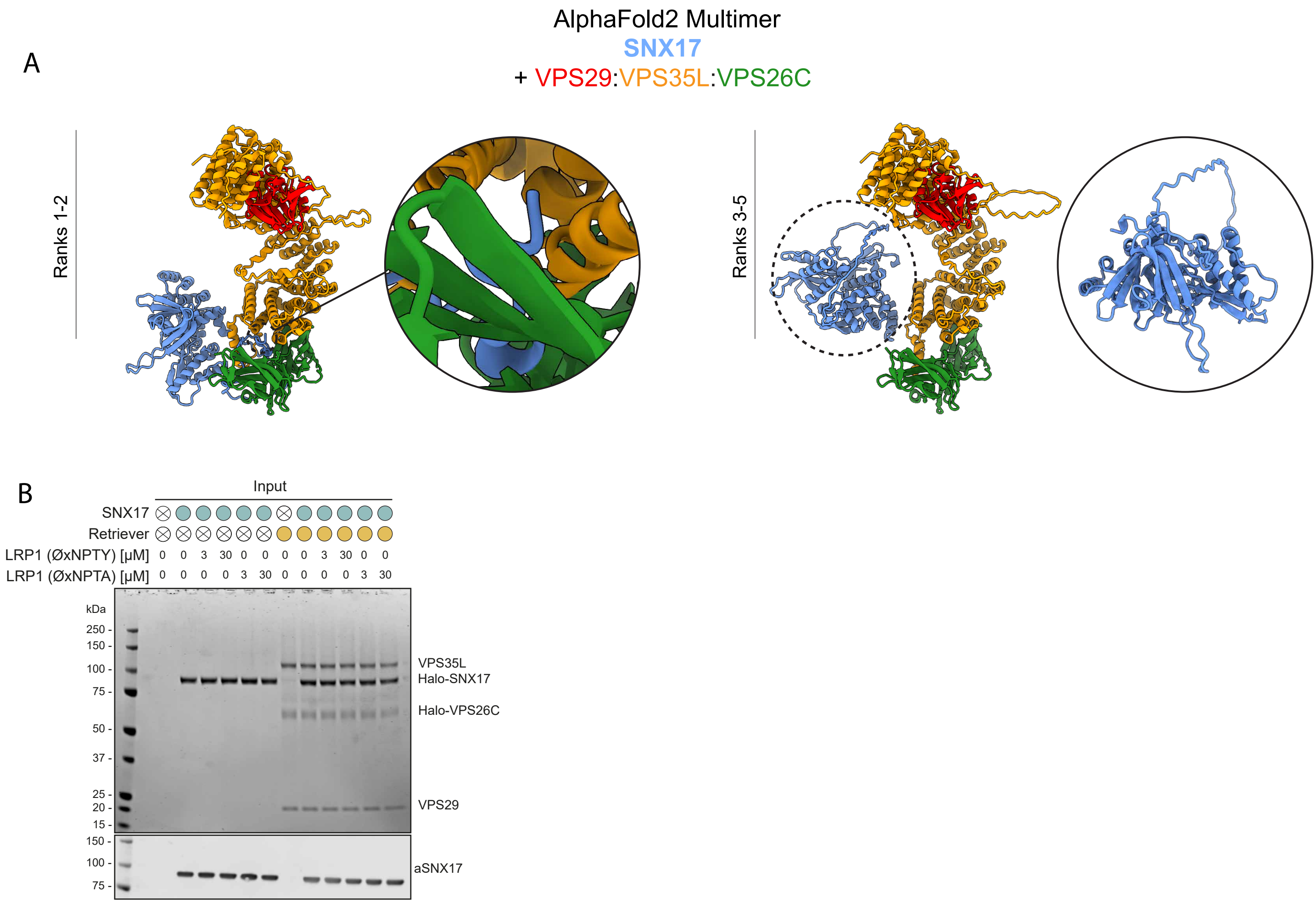
A
AlphaFold2 Multimer
SNX17
+ VPS29:VPS35L:VPS26C
Ranks 1-2
Ranks 3-5
B
Input
SNX17
Retriever
LRP1 (ØxNPTY) [µM] 0 0 3 30 0 0 0 0 3 30 0 0
LRP1 (ØxNPTA) [µM] 0 0 0 0 3 30 0 0 0 0 3 30
kDa
250 -
150 -
100 -
75 -
50 -
37 -
25 -
20 -
15 -
VPS35L
Halo-SNX17
Halo-VPS26C
VPS29
150 -
100 -
75 -
aSNX17

Supplementary Figure 3.

(A) AlphaFold2 was used to predict the binding of full-length SNX17 to the VPS35L-VPS26C interface. Ranks were automatically assigned to the 5 predicted models, with ranks 1 and 2 predicting the interaction between the unstructured C-terminus of SNX17 and VPS35L-VPS26C interface of the Retriever complex, and ranks 3-5 predicting no binding between SNX17 and Retriever and showing unbound, monomeric SNX17. (B) Input proteins for Fig. 5D.

A

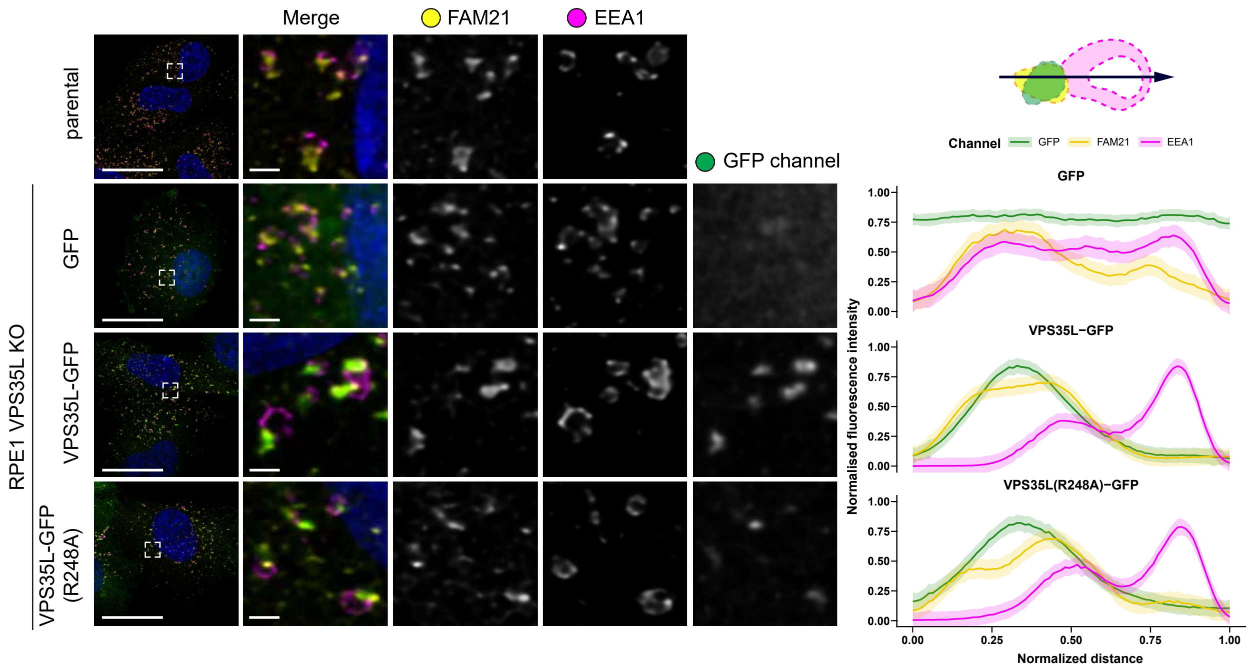

Merge
FAM21
EEA1
parental
GFP channel
RPE1 VPS35L KO
GFP
VPS35L-GFP
VPS35L-GFP (R248A)
Channel
GFP
FAM21
EEA1
GFP
VPS35L-GFP
VPS35L(R248A)-GFP
Normalised fluorescence intensity
Normalized distance
0.00
0.25
0.50
0.75
1.00

B

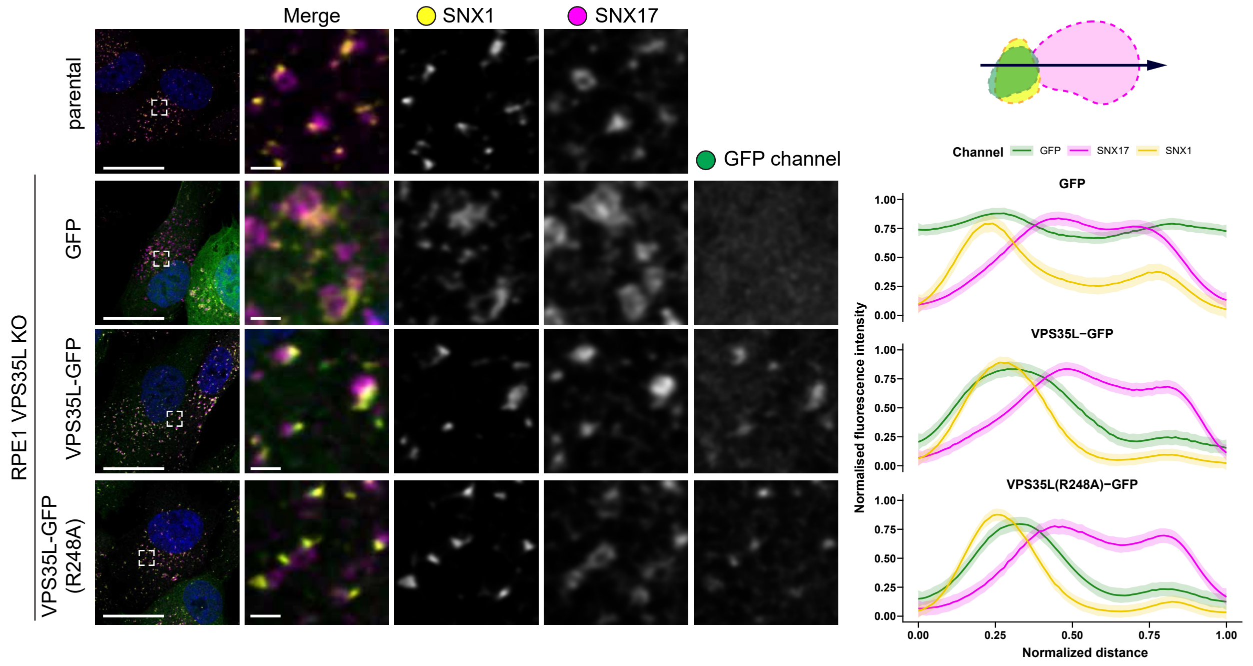

Merge
SNX1
SNX17
parental
GFP channel
RPE1 VPS35L KO
GFP
VPS35L-GFP
VPS35L-GFP (R248A)
Channel
GFP
SNX17
SNX1
GFP
VPS35L-GFP
VPS35L(R248A)-GFP
Normalised fluorescence intensity
Normalized distance
0.00
0.25
0.50
0.75
1.00

Supplementary Figure 4. Retriever complex colocalises with the markers of the WASH and ESCPE-1 complexes.

(A-B) VPS35L KO RPE1 cells were lentivirally transduced with GFP, VPS35L-GFP or VPS35L-GFP(R248A). The localisation of GFP or GFP-tagged proteins was compared to the localisation of endogenous endosome markers EEA1 and FAM21 (WASH complex subunit) (A) or SNX17 and SNX1 (ESCPE-1 subunit) (B). Representative confocal microscopy images are shown. The relative distributions of endosomal markers were evaluated in ImageJ by generating fluorescence intensity line profiles. Line profiles of 30 endosomes from 3 independent experiments were analysed in Rstudio, where the lengths of line scans and raw fluorescence intensities were normalised and averaged. The average profiles are shown on the right. The shading corresponds to the 95% confidence interval. Scale bars shown for full image or inset correspond to 20 µm and 2 µm, respectively.

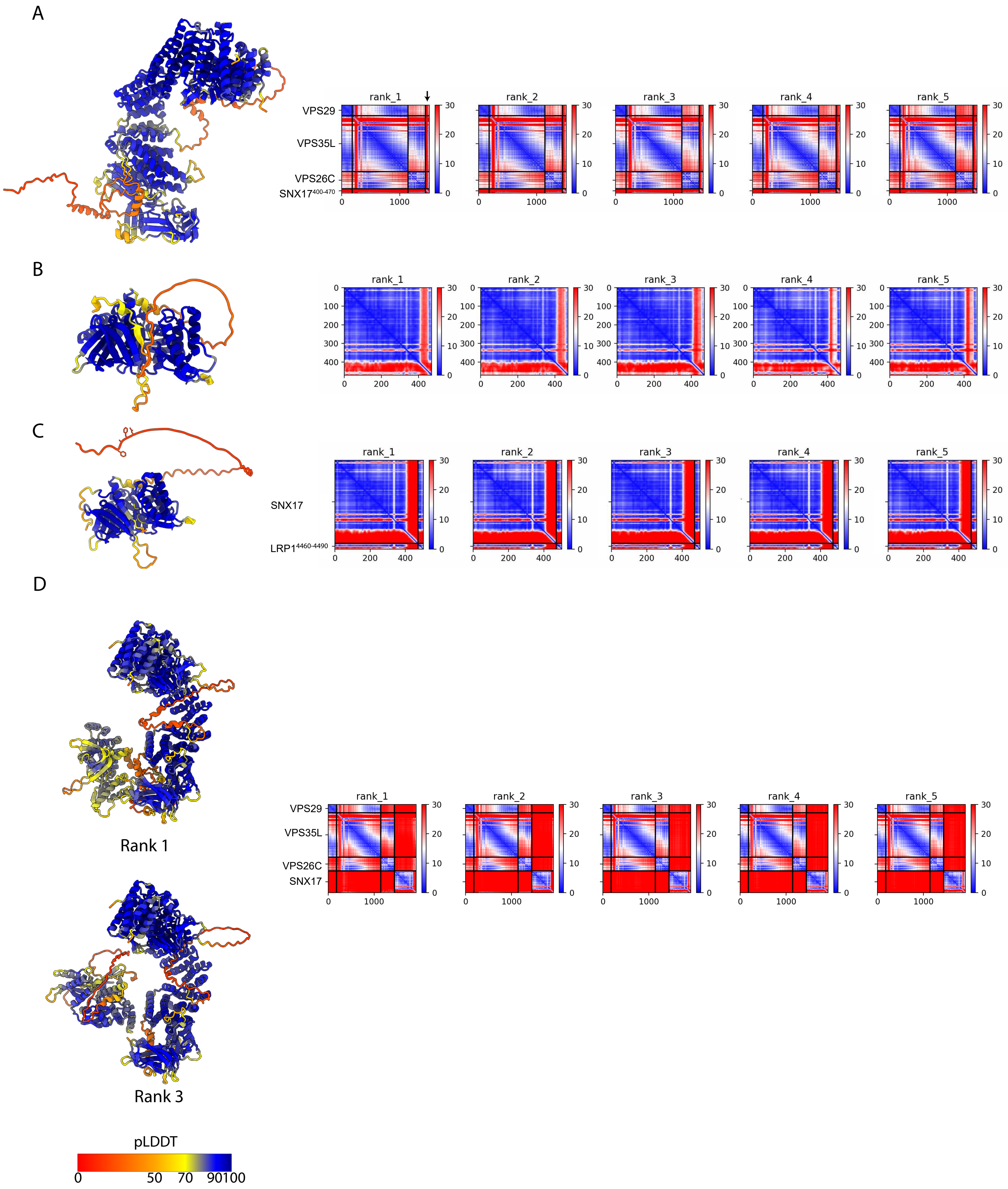

A
VPS29
VPS35L
VPS26C
SNX17400-470
rank_1
rank_2
rank_3
rank_4
rank_5
B
C
SNX17
LRP14460-4490
D
Rank 1
Rank 3
pLDDT
0
50
70
90100

Supplementary Figure 5. AlphaFold2 models and associated predicted alignment error plots.

(A) AlphaFold2 prediction of Retriever (VPS35L, VPS26C, VPS29) and residues 400 to 470 of SNX17. (B) SNX17 full-length. (C) SNX17 full-length modelled against the LRP1 carboxy-tail. (D) SNX17 full-length modelled with Retriever: Rank 1 showing SNX17 carboxy-terminal tail binding to the VPS26C:VPS35L interface, and Rank 3 showing autoinhibited SNX17, not bound to Retriever. Each structure model is coloured by pLDDT score, a measure of confidence where blue is a more confident prediction. Each structure is also accompanied by the predicted alignment error (PAE) plot for each of the five models. PAE plots show the correlation between any given residue in angstroms (Å). Blue indicates that two residues are highly correlated while red indicates no correlation.

Supplementary Table 1. ITC data of cargo peptide binding to full length SNX17 and SNX17ΔC

Interaction	KD (μM)	ΔH (kcal/mol)	ΔG (kcal/mol)	-TΔS (kcal/mol)
SNX17 Full length + LRP1	14.30 ± 0.29	-4.5 ± 0.31	-6.28 ± 0.01	-1.78 ± 0.31
SNX17ΔC + LRP1	4.60 ± 0.11	-4.50 ± 0.11	-6.92 ± 0.01	-2.43 ± 0.11
SNX17 Full length + APP	16.00 ± 0.50	-11.2 ± 0.12	-6.22 ± 0.02	5.03 ± 0.11
SNX17ΔC + APP	7.03 ± 0.50	-10.8 ± 0.39	-6.68 ± 0.04	4.15 ± 0.44
SNX17ΔC + SNX17 tail	189 ± 6.89	-3.96 ± 0.69	-4.83 ± 0.02	-0.86 ± 0.68
SNX17 FL + SNX17 tail	No binding			
SNX17ΔC + LRP1+ SNX17 tail	No binding			

UNCROPPED GELS AND BLOT SCANS FOR SUPPLEMENTARY FIGURES

S1A

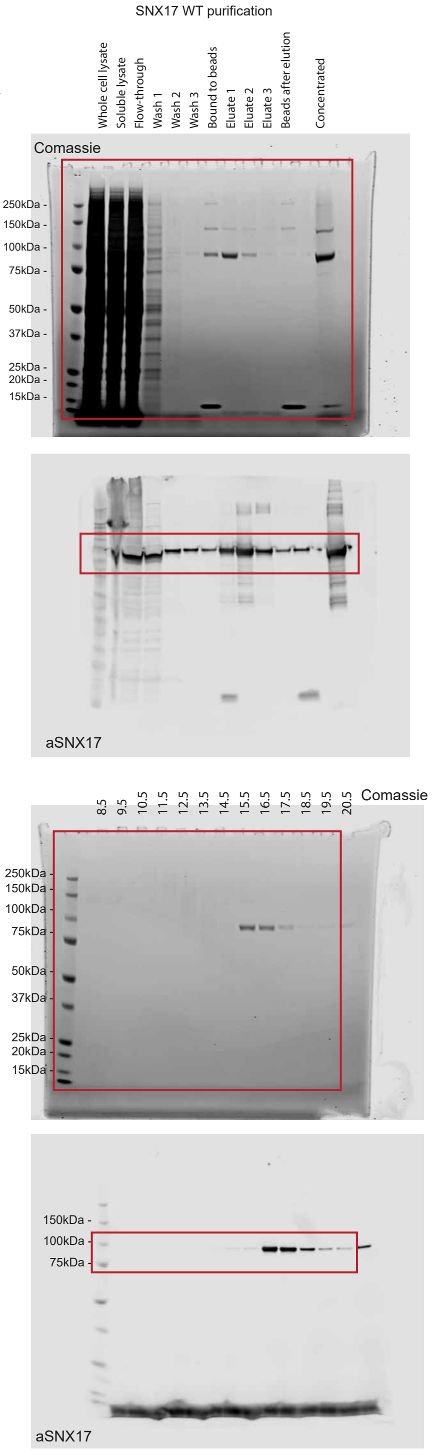
SNX17 WT purification
Whole cell lysate
Soluble lysate
Flow-through
Wash 1
Wash 2
Wash 3
Bound to beads
Eluate 1
Eluate 2
Eluate 3
Beads after elution
Concentrated
Comassie
250kDa -
150kDa -
100kDa -
75kDa -
50kDa -
37kDa -
25kDa -
20kDa -
15kDa -
aSNX17
8.5
9.5
10.5
11.5
12.5
13.5
14.5
15.5
16.5
17.5
18.5
19.5
20.5
Comassie
250kDa -
150kDa -
100kDa -
75kDa -
50kDa -
37kDa -
25kDa -
20kDa -
15kDa -
150kDa -
100kDa -
75kDa -
aSNX17

S1B

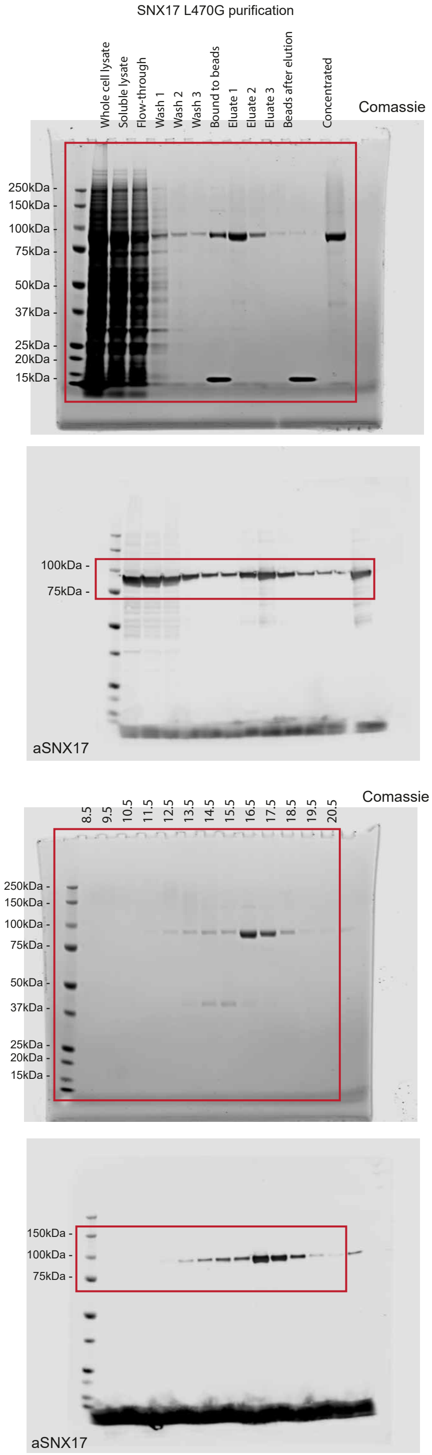
SNX17 L470G purification
Whole cell lysate
Soluble lysate
Flow-through
Wash 1
Wash 2
Wash 3
Bound to beads
Eluate 1
Eluate 2
Eluate 3
Beads after elution
Concentrated
Comassie
250kDa -
150kDa -
100kDa -
75kDa -
50kDa -
37kDa -
25kDa -
20kDa -
15kDa -
100kDa -
75kDa -
aSNX17
8.5
9.5
10.5
11.5
12.5
13.5
14.5
15.5
16.5
17.5
18.5
19.5
20.5
Comassie
250kDa -
150kDa -
100kDa -
75kDa -
50kDa -
37kDa -
25kDa -
20kDa -
15kDa -
150kDa -
100kDa -
75kDa -
aSNX17

S2C

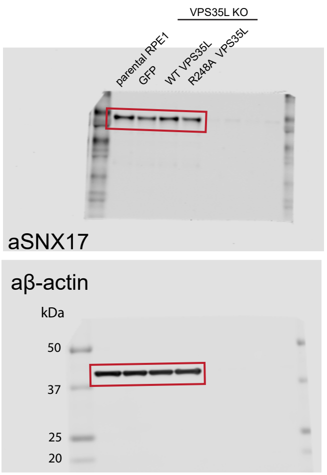
VPS35L KO
parental RPE1
GFP
WT VPS35L
R248A VPS35L
aSNX17
aβ-actin
kDa
50
37
25
20

S2D

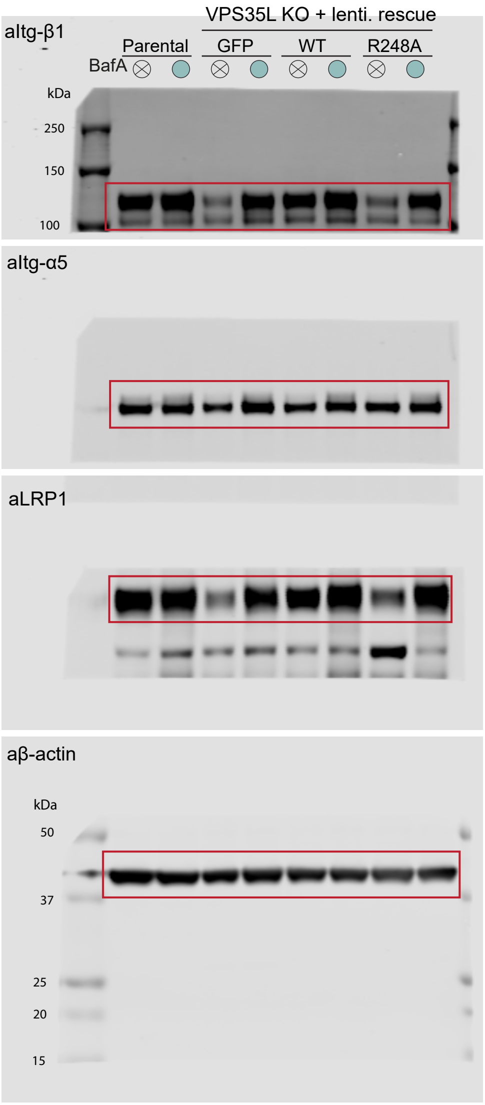
aItg-β1
VPS35L KO + lenti. rescue
Parental
GFP
WT
R248A
BafA
kDa
250
150
100
aItg-α5
aLRP1
aβ-actin
kDa
50
37
25
20
15

S3B

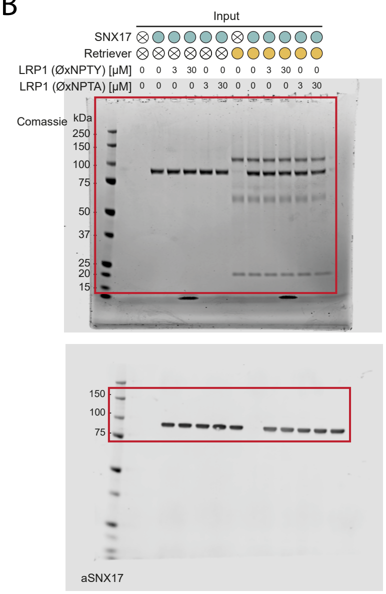
Input
SNX17
Retriever
LRP1 (ØxNPTY) [µM] 0 0 3 30 0 0 0 0 3 30 0 0
LRP1 (ØxNPTA) [µM] 0 0 0 0 3 30 0 0 0 0 3 30
Comassie
kDa
250
150
100
75
50
37
25
20
15
150
100
75
aSNX17
