# Supplementary material for: Mechanism and regulation of cargo entry into the Commander endosomal recycling pathway
Source: Nat Commun. 2024 Aug 21;15:7180. doi: 10.1038/s41467-024-50971-0 (PMC11339278; doi:10.1038/s41467-024-50971-0)
Supplement: Supplementary file 3 — Description of Additional Supplementary Files [file 41467_2024_50971_MOESM3_ESM.pdf]

File name: Supplementary Data 1

Description: List of DNA primers. This file contains a list of primers that were used in this study.
