# Supplementary material for: Mechanism and regulation of cargo entry into the Commander endosomal recycling pathway
Source: Nat Commun. 2024 Aug 21;15:7180. doi: 10.1038/s41467-024-50971-0 (PMC11339278; doi:10.1038/s41467-024-50971-0)
Supplement: Supplementary file 6 — Source Data [file 41467_2024_50971_MOESM6_ESM.zip › Source Data_Gels and Blots.pdf]

1A

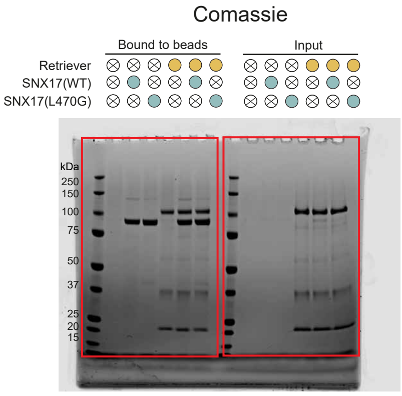
Comassie
Bound to beads
Input
Retriever
SNX17(WT)
SNX17(L470G)
kDa
250
150
100
75
50
37
25
20
15

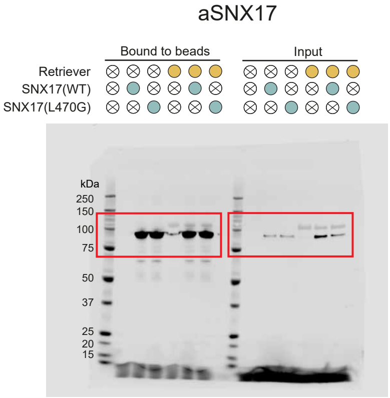
aSNX17
Bound to beads
Input
Retriever
SNX17(WT)
SNX17(L470G)
kDa
250
150
100
75
50
37
25
20
15

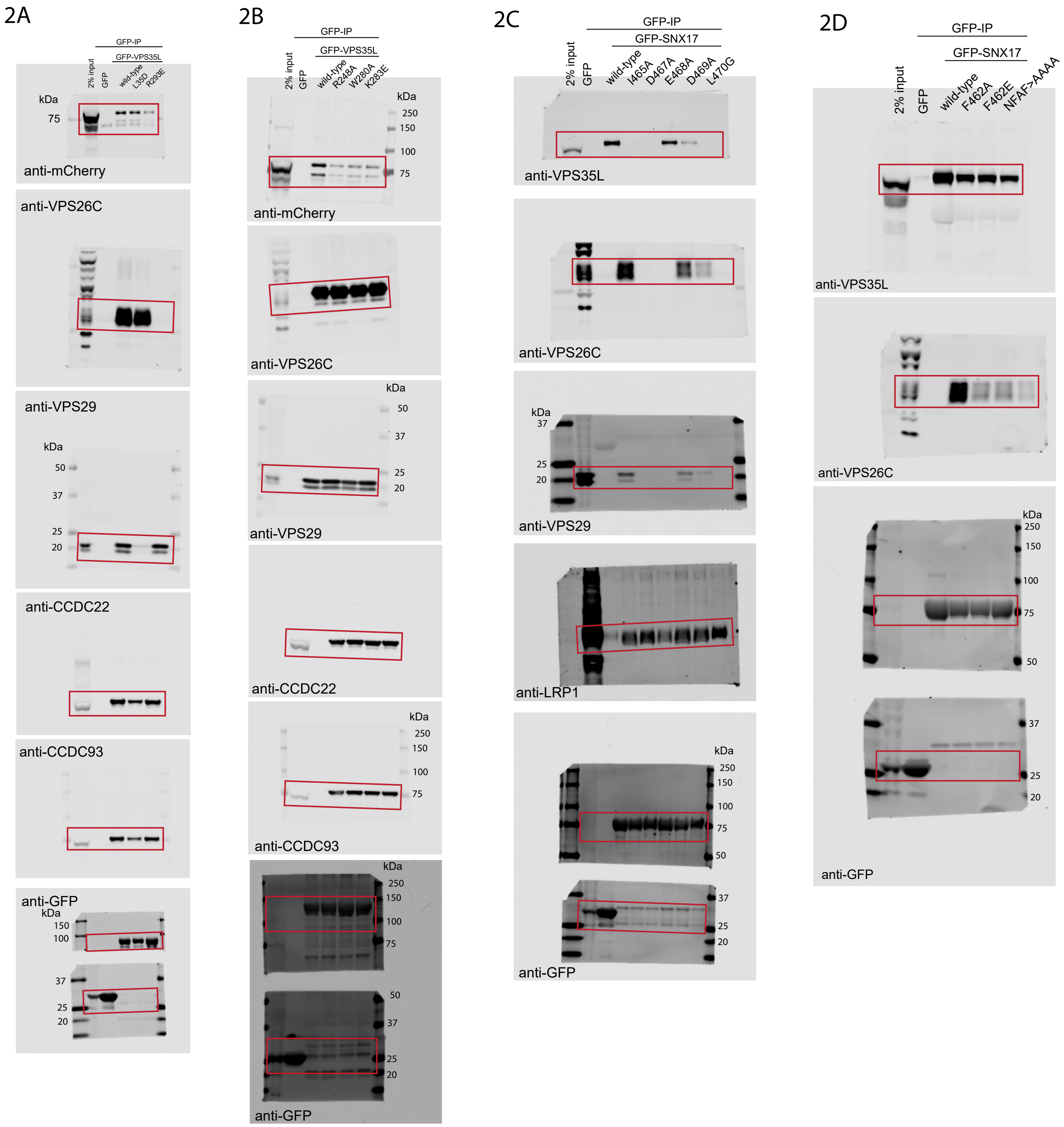
2A
GFP-IP
GFP-VPS35L
2% input
GFP
wild-type
L35D
R293E
kDa
75
anti-mCherry
anti-VPS26C
anti-VPS29
kDa
50
37
25
20
anti-CCDC22
anti-CCDC93
anti-GFP
kDa
150
100
37
25
20
2B
GFP-IP
GFP-VPS35L
2% input
GFP
wild-type
R248A
W280A
K283E
kDa
250
150
100
75
anti-mCherry
anti-VPS26C
kDa
50
37
25
20
anti-VPS29
anti-CCDC22
kDa
250
150
100
75
anti-CCDC93
kDa
250
150
100
75
50
37
25
20
anti-GFP
2C
GFP-IP
GFP-SNX17
2% input
GFP
wild-type
I465A
D467A
E468A
D469A
L470G
anti-VPS35L
anti-VPS26C
kDa
37
25
20
anti-VPS29
anti-LRP1
kDa
250
150
100
75
50
37
25
20
anti-GFP
2D
GFP-IP
GFP-SNX17
2% input
GFP
wild-type
F462A
F462E
NFAF>AAAA
anti-VPS35L
anti-VPS26C
kDa
250
150
100
75
50
37
25
20
anti-GFP

3C

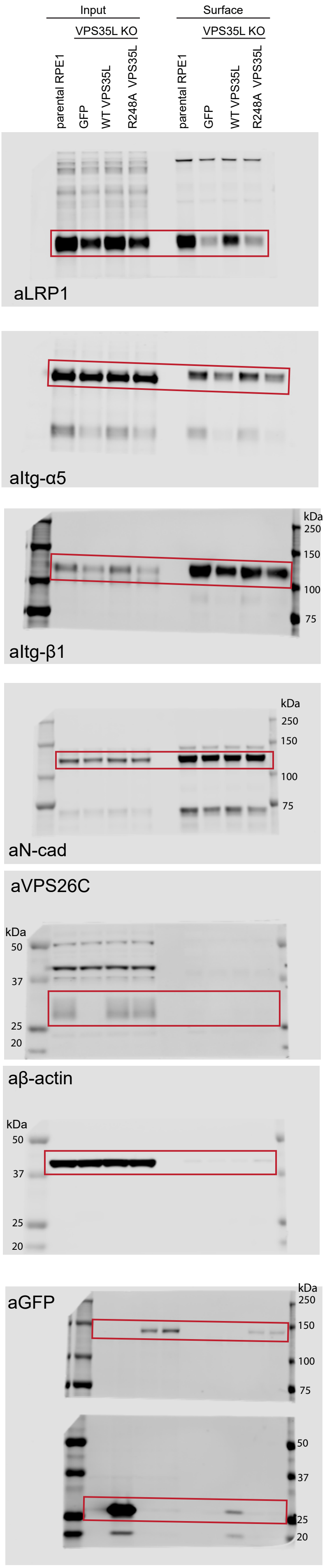

Input
Surface
VPS35L KO
VPS35L KO
parental RPE1
GFP
WT VPS35L
R248A VPS35L
parental RPE1
GFP
WT VPS35L
R248A VPS35L
aLRP1
aItg-α5
aItg-β1
kDa
250
150
100
75
aN-cad
kDa
250
150
100
75
aVPS26C
kDa
50
37
25
20
aβ-actin
kDa
50
37
25
20
aGFP
kDa
250
150
100
75
50
37
25
20

4C

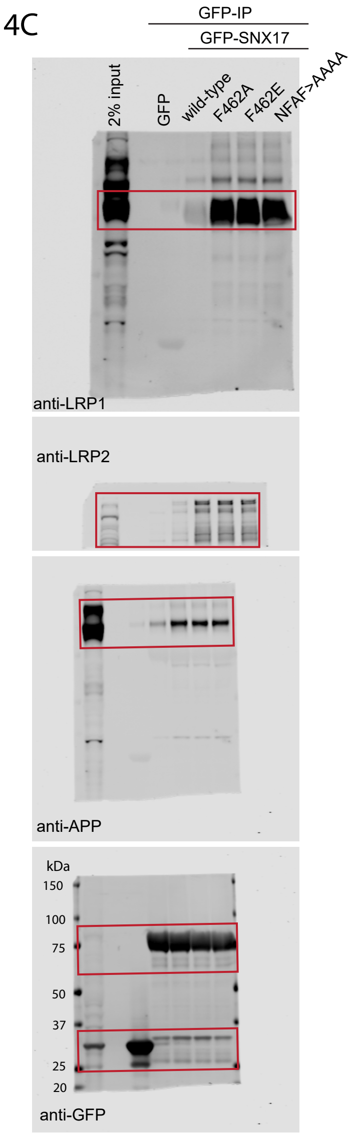

GFP-IP
GFP-SNX17
2% input
GFP
wild-type
F462A
F462E
NFAF>AAAA
anti-LRP1
anti-LRP2
anti-APP
kDa
150
100
75
50
37
25
20
anti-GFP

4D

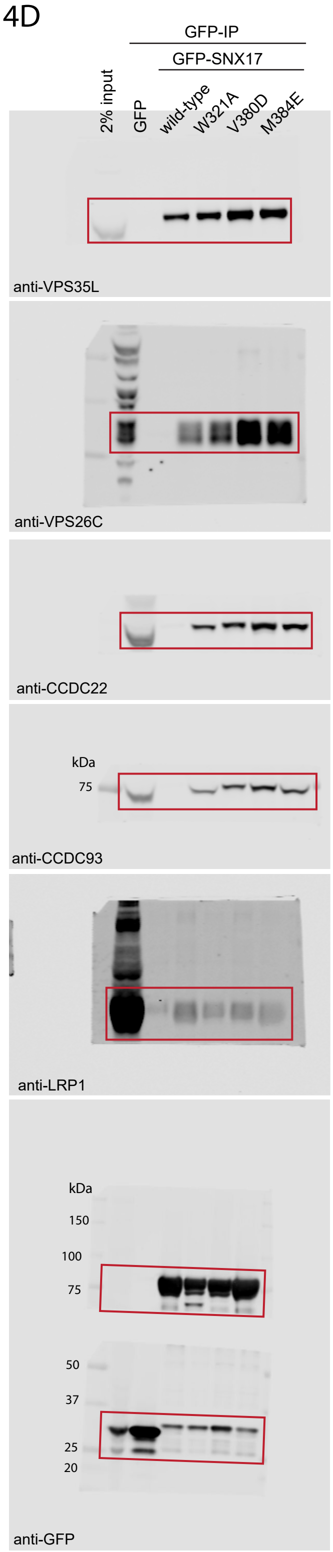

GFP-IP
GFP-SNX17
2% input
GFP
wild-type
W321A
V380D
M384E
anti-VPS35L
anti-VPS26C
anti-CCDC22
kDa
75
anti-CCDC93
anti-LRP1
kDa
150
100
75
50
37
25
20
anti-GFP

5D

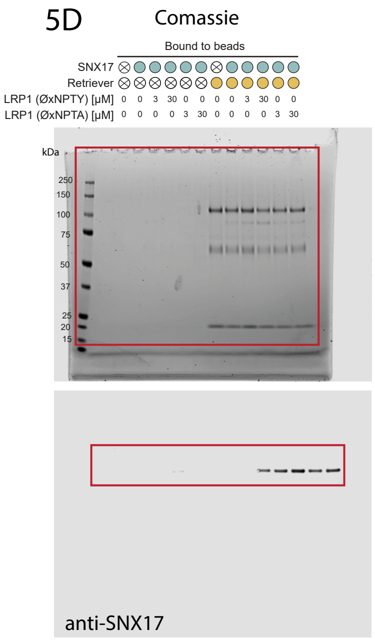
Comassie
Bound to beads
SNX17
Retriever
LRP1 (ØxNPTY) [µM] 0 0 3 30 0 0 0 0 3 30 0 0
LRP1 (ØxNPTA) [µM] 0 0 0 0 3 30 0 0 0 0 3 30
kDa
250
150
100
75
50
37
25
20
15
anti-SNX17
